# Tannin and Glycerine

**Published:** 1872-06

**Authors:** R. Rother


### SELECTIONS

### Tannin and Glycerine.

BY R. ROTHER.

Tannic acid is frequently prescribed in concentrated solution with glycerine; but tannin, commercially obtained, possesses various impurities which either remain as insoluble turbidity or discolor the solution. Firstly, a green resinous coloring-matter, insoluble in water but soluble in strong alcohol and glycerine, invariably occurs. This contamination results from the solvent action of the ether in the original process of extracting the tannin. Secondly, metallic chips of copper, iron, etc., from the vessels in which the tannin was dried, are never absent.

A concentrated solution of tannin is nearly indispensable among the requisites of the prescription department. An aqueous solution, however concentrated it may be, will spoil. An alcoholic solution is often objectionable, but an aqueous solution, containing glycerine, can be utilized on almost all occasions.

The solution is best adjusted by weight; it is perfectly stable, clear, and transparent, and contains one troy ounce of tannic acid in two troy ounces of the solution, that is, half tannin by weight. The solvent is the other half, or $\frac{1}{4}$ each by weight, glycerine and water. More than this proportion of glycerine cannot be used to advantage, as the liquid becomes too thick to pour conveniently. This solution cannot be prepared, however, by directly combining the three ingredi ents, as the impurities must first be removed; and the only preliminary solvent for this purpose, which the writer has found to answer perfectly, is a mixture of equal measures of strong alcohol and water. A very concentrated solution, in the proportion of two parts of liquid to one of tannin, can be formed with the aid of heat, which filters with the greatest facility, leaving the resinous coloring matter and the metals untouched.

Alcohol alone, in the proportion of four to one of tannin, would not filter well. Water, in the proportion of at least four to one of tannin, would not filter even as rapidly as the solution with alcohol, and whilst the alcoholic solution becomes turbid with water, the aqueous solution becomes clear from the first, and moreover was always much darkened by the metallic impurities forming colored soluble tannates. The preliminary solvent and permanent solvent above proposed are therefore the only available ones. These form a light green, thin, syrupy solution, miscible with glycerine and water in all proportions without losing their brightness, and forming in a more dilute condition colorless solutions.

From these observations the following formula is deduced:

| | | |
|---|---|---|
| Take of Tannin | 8 | troy ounces. |
| Glycerine | 4 | " " |
| Strong alcohol | 8 | fluid ounces. |
| Water | 8 | " " |

Mix the alcohol and water; add the tannin, and apply heat until the tannin has dissolved. Filter hot, then add the glycerine, and evaporate by a careful heat until the solution weighs 16 troy ounces.—*The Pharmacist*, Dec., 1871.

